# Direct 3D Printing of Silica Doped Transparent Magnesium Aluminate Spinel Ceramics

**DOI:** 10.3390/ma13214810

**Published:** 2020-10-28

**Authors:** John M. Pappas, Xiangyang Dong

**Affiliations:** Mechanical and Aerospace Engineering, Missouri University of Science and Technology, Rolla, MO 65409, USA; jmpn47@mst.edu

**Keywords:** transparent ceramics, magnesium aluminate spinel, silica doping, laser direct deposition, Additive manufacturing

## Abstract

Transparent magnesium aluminate spinel ceramics were additively manufactured via a laser direct deposition method in this study. With a minimum porosity of 0.3% achieved, highly transparent spinel samples with the highest total optical transmittance of 82% at a wavelength of 632.8 nm, were obtained by a 3D printing approach. However, cracking was found to be a major issue affecting printed spinel samples. To control prevalent cracking, the effect of silica dopants was investigated. Increased silica dopants reduced average total crack length by up to 79% and average crack density by up to 71%. However, a high dopant level limited optical transmission, attributed to increased porosity and formation of secondary phase. Further investigation found that with decreased average fracture toughness, from 2.4 $\mathrm{MPa}\cdot m^{1/2}$ to 1.9 $\mathrm{MPa}\cdot m^{1/2}$, the obvious reduction in crack formation after doping was related to decreased grain size and introduction of softer secondary phase during deposition. The study demonstrated the feasibility of the proposed laser direct deposition method in directly fabricating transparent spinel ceramics while dopants showed potentials in addressing cracking issues.

## 1. Introduction

Transparent ceramics are of great interest for a variety of applications including transparent armor [1,2] as well as windows and domes for aircraft and defense [3,4,5]. The most prominent candidates include magnesium aluminate spinel (MgAl_2_O_4_, also referred to as “spinel”), aluminum oxynitride (ALON), and sapphire. Cubic-polycrystalline transparent ceramics like spinel and ALON have several distinct advantages over non-cubic ceramics like sapphire (alumina). Non-cubic transparent ceramics are birefringent in nature [6]. Fabrication of sapphire not only is very costly [7,8] but also presents greater difficulty in producing large or complex structures due to the necessity of producing single crystal to obtain transparency [9]. Fabrication of polycrystalline alumina presents its own challenges, requiring grains smaller than one-tenth the optical wavelength of interest to achieve transparency [6,10,11]. In contrast, cubic-polycrystalline transparent ceramics including spinel and ALON do not exhibit birefringence. Spinel powders are more widely available and have a wide range of solubility at elevated temperature, especially for alumina [12,13]. Solid solution spinel is typically represented as MgO·nAl_2_O_3_, and the useful range for transparent ceramics is typically 0.98 < *n* < 3 [14,15]. Spinel is also highly desirable for excellent transparency to electromagnetic radiation at a wider range of wavelengths [16] compared to ALON. Besides high hardness, excellent chemical resistance, and good thermal shock resistance [17], the lowest density among all other transparent ceramics makes spinel useful for lightweight armor applications [18].

Traditional manufacturing methods for transparent spinel ceramics typically include hot pressing and sintering followed by hot isostatic pressing (HIP) to reach full densification [19]. With this method, infrared domes and radomes can be fabricated but typically require substantial polishing to the desired shape, greatly adding to manufacturing costs [20]. Spark plasma sintering (SPS) was proposed [10,21,22] to shorten processing times and reduce grain sizes of transparent spinel parts [9], and significant contributions have been made to optimize parameters for SPS of transparent spinel ceramics [23]. However, fabrication of complex shapes poses additional challenges to SPS due to the necessity of compaction using graphite dies [24]. In comparison, additive manufacturing (AM) techniques have potentials to simplify fabrication of near-net shape, complex spinel parts of near full densification, largely reducing time and costs related to post-processing including HIP, machining [25], and polishing.

Transparent ceramics only exhibit the highest transparency when residual porosity and process-induced cracking are minimized. Our recent studies [26] showed that porosity was one important factor limiting the transparency of additively manufactured spinel samples by laser direct deposition. Laser processing conditions, particularly laser power and powder flow rate during laser deposition process, were found to have significant effects on the porosity reduction. Yan et al. [27] found that ultrasonic vibration contributed to reduction in porosity for (nontransparent) alumina-zirconia eutectic ceramics. It was attributed to acoustic flow of the melt aiding the natural buoyant effect of gas bubbles, allowing more to escape before solidification. Traditional sintering-based methods used sintering dopants to reduce porosity and promote densification of transparent spinel ceramics. LiF is commonly used as a sintering aid for traditionally manufactured spinel ceramics to reach the highest transparency [12]. On the other hand, doping with rare earth ions including Dy^3+^ and Tb^3+^ is useful to increase certain luminescence bands in spinel ceramics, which may ultimately be used as emitting medium and optical radiation converters [28]. However, very few studies have been done to investigate the doping effects on residual porosity and transparency of additively manufactured transparent ceramics.

On the other hand, dopants showed positive effects in controlling crack formation during laser direct deposition of nontransparent ceramics. Niu et al. [29] showed that second phase doping of alumina with yttrium aluminum garnet (YAG) and zirconia both significantly reduced cracking in laser direct deposited ceramics. Single-bead walls prepared with alumina/YAG had notably reduced cracking, and cracks were completely eliminated in alumina/zirconia parts at the eutectic ratio as a result of significant microstructural refinement. In another study, Niu et al. [30] showed the crack suppression effect of TiO_2_ dopant on Al_2_O_3_/Al_2_TiO_5_ composites fabricated by laser direct deposition. A mismatch in the coefficient of thermal expansion (CTE) resulted in compressive residual stresses in the resultant Al_2_TiO_5_ matrix. It promoted crack deflection and crack pinning, both of which were conducive to consumption of crack propagation energy.

The reduction in crack formation within laser direct deposited ceramics is closely related to the obtained mechanical properties after the introduction of dopants. The addition of zirconia to alumina had great effects on both the microstructure and mechanical properties of deposited ceramics [31,32]. Microhardness increased due to grain refinement and precipitation hardening. Fracture toughness monotonously increased as a result of transformation toughening and cracking mechanisms including crack bridging, branching and deflection. Similarly, Wu et al. [33] found that TiO_2_ doping in Al_2_O_3_/Al_2_TiO_5_ composites resulted in a maximum of 30% improvement in fracture toughness over pure alumina at a relatively low dopant percentage. Liu et al. [34] also showed improved fracture toughness for eutectic alumina/zirconia ceramics prepared by laser direct deposition. It was proposed to be caused by alternating residual stress fields formed during cooling due to a mismatch of thermal expansion coefficients, which resulted in crack bridging and deflection during crack propagation. However, it is not clear how dopants will affect laser direct deposited transparent ceramics, in particular crack formation.

Although there are numerous studies on AM of ceramics, very few studies have been done to address laser-based AM of transparent ceramics. Direct ink writing (DIW) process was recently applied in fabrication of transparent yttrium aluminum garnet (YAG) [35]. While DIW has the potential to create complex ceramic shapes, it is necessary to use binders and extensive consolidation/sintering procedures including cold isostatic pressing, binder removal, vacuum sintering, and HIP, all of which increase processing time and costs. Without HIP, no transparency was obtained for the spinel parts printed by a similar extrusion-based method [36].

In this study, we attempt to address the knowledge gap in laser-based AM of transparent spinel ceramics. The feasibility of laser direct deposition was studied by direct 3D printing of transparent spinel ceramics with dopants. It is the first study showing that with a great reduction of porosity, transparent spinel ceramics were additively fabricated by laser direct deposition. Cracking was shown to be a major limiting factor for the proposed 3D printing method. Inspired by the positive effects of dopants in crack control [32,34,37], its effects on laser direct deposition of transparent spinel ceramics were thus explicitly investigated. Consistent with the previous studies [29,33,38], the process of using dopants/additives was also defined as “doping” to study the effects of additives on laser direct deposited ceramics and to further design laser direct deposited ceramics. Silica was selected in this study due to its low CTE [39] and potentials to lower thermal stress and reduce crack formation during deposition process. The obtained sample morphology, microstructure, composition, and mechanical properties were systematically investigated in terms of doping percentages.

## 2. Materials and Methods

### 2.1. Materials

Alumina-rich spinel powders (MgO·1.4Al_2_O_3_, AR78-90MY by Almatis, Leetsdale, PA, USA) were used in this study. A purity of approximately 99.4% with detailed compositions [40] and an average particle size of 22.8 µm were reported by the vendor. Our preliminary studies [41] showed that cracking was one of the two main factors that hindered the transparency of the printed spinel samples. Alumina-rich spinel was thus selected to minimize cracking due to its higher fracture toughness than its stoichiometric counterparts [42]. Meanwhile, previous studies showed that an alumina rich spinel composition resulted in higher transmittance values over a broad range of wavelengths than stoichiometric or near stoichiometric spinel [15,43,44].

Prior to fabrication, powder agglomerates were broken up by passing through a No. 325 mesh sieve with 44 µm opening size. Powder flowability was significantly influenced by moisture content of spinel powders. Hence, the powder was heated in air to 200 °C for at least eight hours. All powders were also kept in an oven at 200 °C to prevent water adsorption prior to deposition. This allowed consistent powder flow throughout experiments. Alumina substrates with dimensions 108 mm × 53 mm × 4 mm were used due to thermal expansion compatibility with deposited spinel ceramics. The use of alumina substrates also helped minimize substrate contamination of deposition, which would reduce spinel part transparency.

Silica dopants (406 Colloidal Silica Adhesive Filler, West System, Bay City, MI, USA) were blended with pure spinel powder with doping compositions varying from 0.5 to 10 wt.%. Spinel-silica mixtures were prepared using a ball mill with alumina milling media to eliminate the possibility of powder contamination and high-purity acetone solvent for two hours to ensure thorough and homogeneous blending. Acetone was then eliminated using a Buchi R124 rotary evaporator (New Castle, DE, USA). Following acetone removal, the powder blends were calcined at 600 °C in a Lindberg furnace (Riverside, MI, USA) for six hours to eliminate any organic contaminants.

### 2.2. Experimental Setup

**Figure 1:**
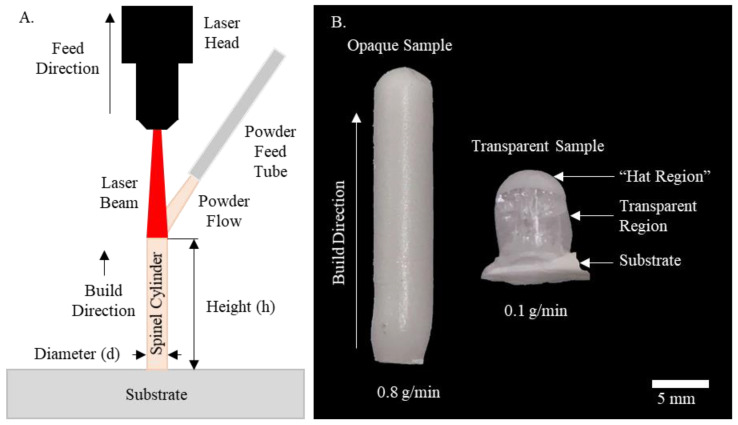
(**A**) Schematics of cylindrical structure deposition process and (**B**) Typical printed pure spinel samples with powder flow rates of 0.8 g/min (left) and 0.1 g/min (right).

The laser direct deposition setup in this study included a 1.7 kW continuous wave mode CO_2_ laser (Convergent Energy Arrow Ultimate Model) operating at 10.6 µm wavelength for its high absorbance by oxide ceramics [45]. Powder delivery was achieved with a single hopper powder feeder (Powder Motions Lab, X2W, Rolla, MO, USA) with feed rate repeatability of less than 1%. Argon (≥99.997% pure, Airgas, Radnor, PA, USA) was used as conveying gas to deliver prepared powder through a powder feed tube into the laser generated melt pool. Figure 1A shows schematics of the deposition process for cylindrical samples used in this study.

During the fabrication process, a continuous cylinder sample was printed perpendicular to the substrate surface. Ceramic powder was continuously fed into the melt pool as the CO_2_ laser displaced in the vertical direction. A laser spot size of 5 mm was used in this study. High laser intensities and temperatures fully melted the delivered powder and hence formed the deposited samples. Immediately after deposition, powder flow was shut off to allow the cylinders to cool in ambient air, without the influence of the conveying gas or powder flow. It is worth noting that the vertical printing strategy was implemented for this study to simply the fabrication process as inspired by the Verneuil method for flame fusion of gemstones [46]. In addition, as suggested by previous studies [47,48], understanding the vertical build approach would facilitate fabrication of freestanding and lattice structures or even internal complex features without support materials. This would be especially beneficial for AM of ceramics. Due to high deposition temperatures and melting point of ceramic materials, support materials would be very difficult to remove.

As shown in Figure 1B, our preliminary studies showed an obvious transition from opaqueness to transparency with a significant reduction of powder flow rate due to porosity reduction [26]. A powder flow rate of 0.1 g/min and a laser power of 580 W were found to yield best transparency and print resolution and thus were used in this study. It should be noted that our previous studies guided selection of the optimal parameters for reduced porosity the printed spinel samples [26].

It was also critical to match the laser head vertical feed rate with the deposition buildup rate to maintain a consistent deposition process. This was achieved through an iterative process. For each parameter set, the vertical feed rate was first approximated based on previous testing, and several samples were then printed with slightly varied rates until the feed distance and cylindrical height converged. A matched vertical feed rate of 0.7 mm/min was found and utilized based on the processing conditions indicated above.

### 2.3. Sample Characterization

To characterize microstructure, the printed samples were first sectioned on a Leco VC-50 low speed diamond saw (St. Joseph, MI, USA) along the planes perpendicular to the build direction to obtain cylindrical samples with an approximate thickness of 2.2 mm. All cross-sections were taken 2 mm from the alumina substrate for comparison and analysis of the bulk spinel. Hence, the potentially detrimental effects of the substrate (such as more rapid cooling and compositional dilution) were minimized. Prior to sectioning, the sample was mounted in an acrylic mounting system (VariDur, Buehler, Bluff, IL, USA) to minimize processing-induced damage and facilitate further post-processing. After sectioning, both sides of the samples were ground and polished using a semi-automatic grinder/polisher (Tegramin-30, Struers, Cleveland, OH, USA) according to ASM standards [49] with progressively finer polishing compounds down to 0.25 μm diamond suspension on a felt pad.

**Figure 2:**
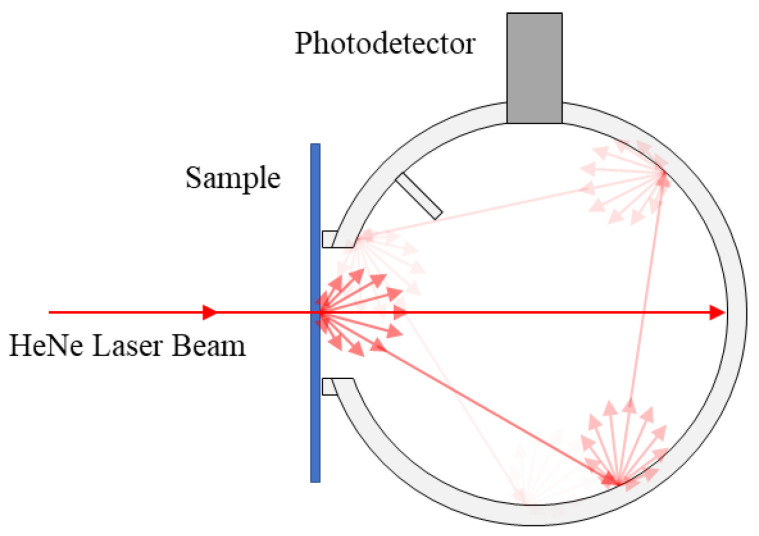
Schematics of total transmittance measurement.

To characterization optical transmission, in-line transmittance was measured on a Varian Cary 300 UV-Visible Spectrophotometer, (Santa Clara, CA, USA). As the majority of the transmission was found to be diffuse, the total transmittance of the polished samples was also measured using an integrating sphere (LabSphere 2525, North Sutton, NH, USA) in Figure 2. The total transmittance of a 0.63 mm diameter HeNe laser beam (λ = 632.8 nm) (Newton, NJ, USA) was calculated as the ratio of the integrated power with the sample to that with no sample (after subtracting the background light).

Optical micrographs of the polished cross-sections were taken with a digital microscope (KH-8700, Hirox, Hackensack, NJ, USA). FEI Quanta 600 FEG Environmental Scanning Electron Microscope (Waltham, MA, USA) was used for scanning electron microscopy (SEM) and energy dispersive X-ray spectroscopy (EDS) using a Bruker Quantax 200 (Oak Park, IL, USA) with XFlash^®^ 6 addon. To avoid electron charging and improve SEM resolution, a 25 nm thick gold-palladium coating was applied to the surface of spinel samples using a Hummer VI sputter coater (Sparks, NV, USA). Crystalline phases of the initial powder and printed spinel samples were examined with powder X-ray diffraction (XRD) analysis. The XRD data was acquired using a PANalytical X’Pert Pro multi-purpose diffractometer (Westborough, MA, USA) with a CuKα radiation source.

Porosity, crack formation, and grain size were all characterized through image analysis of obtained cross-sectional images. FIJI image analysis software [50] was used to binarize and threshold images to only show pores and cracks, respectively. The built-in particle analysis function was used to determine the porosity area percentage. Crack formation was characterized by total crack length, average crack length, and crack density. Crack length was first measured by a ridge detection plugin [51]. The total crack length was calculated based on the summation of the length of all cracks present on the cross-sectional surface, while average crack length was the total crack length divided by the number of cracks. Crack density was further obtained through dividing the total crack length by the cross-sectional area of the sample analyzed. Calculated equivalent circular diameters [52] were used to characterize grain size and were obtained from optical microscope images: the images were first post-processed in the FIJI image analysis software to determine the area of each individual grain; based on these data, average grain area was then calculated and converted to equivalent circular diameter.

Microhardness measurements were taken with a Vickers indentation machine (Struers Duramin-5, Cleveland, OH, USA). The reported hardness values were an average of 10 admissible indentations. Indentations were made with a 9.8 N load maintained for 10 s on the polished sample cross-section. Fracture toughness was calculated by measuring the length of Palmqvist cracks originating at the indenter diagonals.3. Results and Discussion

## 3. Results and Discussion

### 3.1. Sample Morphology and Optical Transmittance

**Figure 3:**
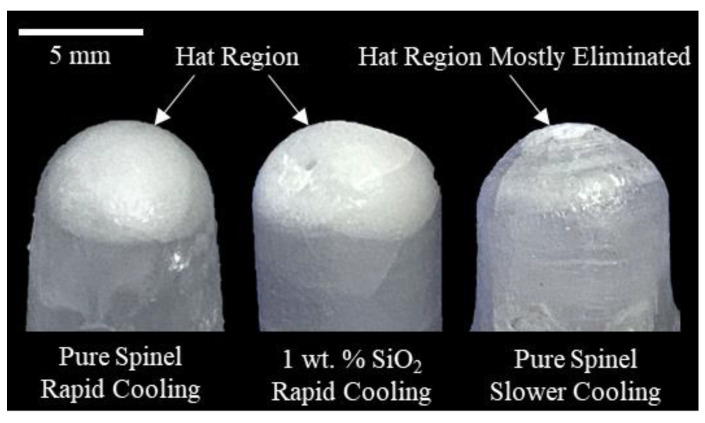
Typical “hat region” observed in both pure and silica doped spinel samples with rapid cooling compared to samples printed with slower cooling after deposition.

As observed in both pure spinel and silica doped samples Figure 3, a common visible defect of the printed cylindrical samples was the presence of a non-transparent region on the top, i.e., “hat region”. The formation of this region was attributed to shrinkage cavities formed during rapid cooling and solidification process after printing [26]. After finishing deposition of last layer and with immediate removal of laser input energy, the surface of the melt rapidly solidified before the central volume, leading to a volumetric constraint. This constraint resulted in insufficient liquid phase replenishment, leading to the formation of shrinkage cavities within the top region. These shrinkage cavities, in the form of porosity, scattered light transmission and deteriorated the transparency of this region, thus forming the hat region. It is possible to minimize or even eliminate the formation of this region by lowering cooling rate after printing. This can be achieved by gradually lowering laser input energy after depositing last layer. As shown in Figure 3, a pure spinel sample with almost no hat region was obtained by gradually reducing laser power at an increment of 40 W every five minutes after deposition.

**Figure 4:**
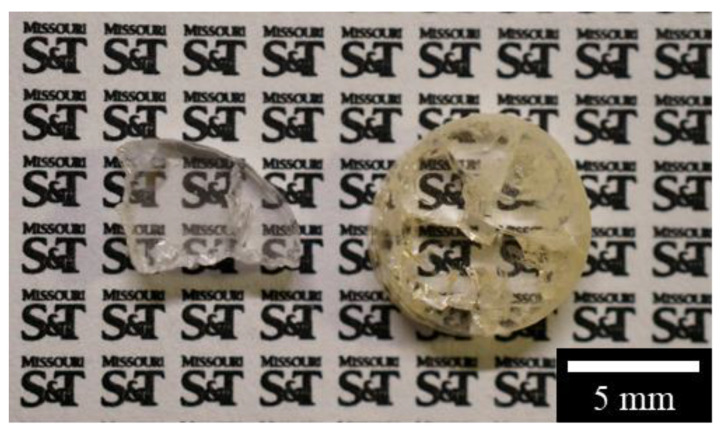
Typical printed pure spinel samples after crossing-sectioning and polishing. Obvious cracking is observed with part of a cracked pure spinel sample also shown.

Another prevalent defect within nearly all printed samples was cracking as noticed in Figure 1B and Figure 3. It was more obviously observed in the cross-sectioned and polished pure spinel samples in Figure 4. A radial crack pattern was observed, indicating the presence of circumferential thermal stress distribution during laser direct deposition of cylindrical structures. Residual cracks scattered light transmission and limited the transparency of certain regions.

**Figure 5:**
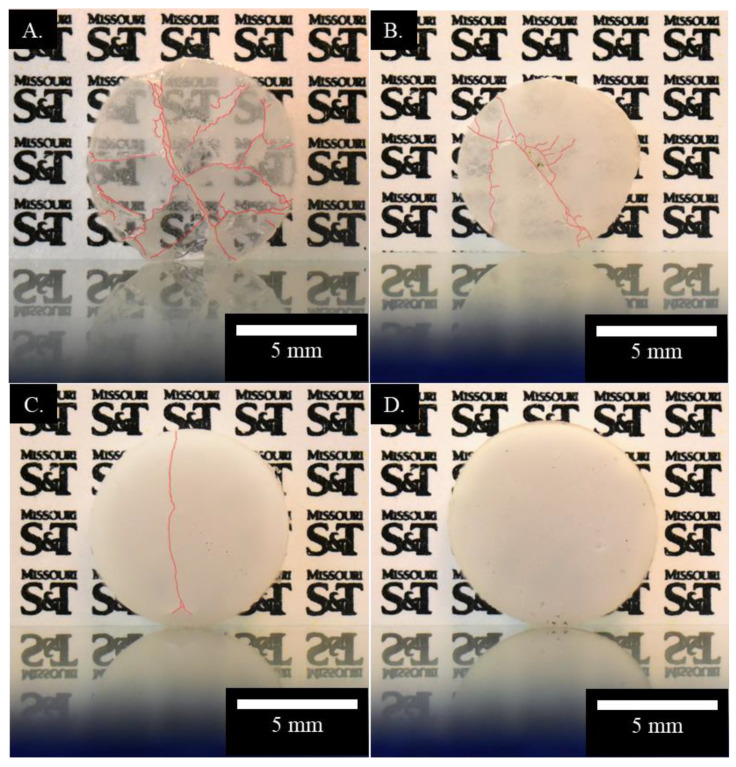
Comparison of cracking (highlighted) and transparency for polished spinel samples printed with silica dopant percentages of (**A**) 0.5 wt.%, (**B**) 3 wt.%, (**C**) 5 wt.%, and (**D**) 10 wt.%.

On the other hand, as highlighted in Figure 5, the addition of silica dopants lowered crack formation within the printed spinel samples. The cracks were even eliminated at one 10 wt.% silica doped sample, showing the potentials of doping in significantly reducing cracks during laser direct deposition of transparent spinel ceramics. However, the obtained transparency severely deteriorated at increasing doping level. Compared to the pure spinel samples in Figure 4, the addition of merely 0.5 wt.% silica dopants lowered the obtained transparency shown in Figure 5A. Further increase of dopants drastically reduced the optical transmission, indicating the need of minimizing the doping level. Although the introduction of dopants during laser direct deposition clearly lowered the crack formation as shown in Figure 5, it will be necessary to understand how to more efficiently control crack formation with a minimal doping level so that high purity transparent spinel ceramics can be fabricated. Thus, detailed investigations on laser direct deposited spinel ceramics were carried out below to further characterize the effects of silica dopants on the microstructure, composition, and mechanical properties. In particular, this study examined residual porosity and secondary phase, the presence of which will increase light scattering due to distinctly different refractive indices compared to that of spinel [18,19,53].

It is also worth noting that despite same processing conditions used, obvious variations in sample diameters and notable irregularities were observed in Figure 4 and Figure 5 for the printed circular shaped samples. This is believed to be affected by high viscosity of ceramics, which hindered uniform spreading of melt pool before solidification [54]. The addition of silica dopants may also alter the melt viscosity. The increased surface roughness of the printed optical parts may increase needs of post-processing and manufacturing costs. Thus, future studies will be performed to understand the thermodynamics during laser melting and solidification process of spinel ceramics.

**Figure 6:**
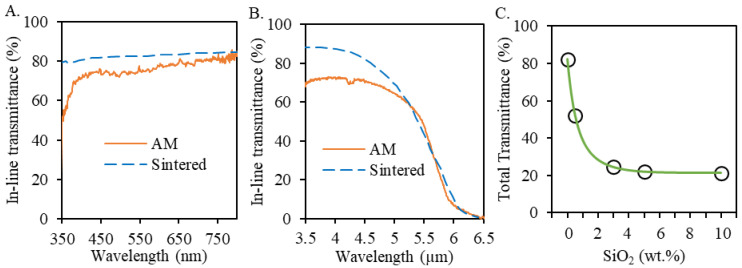
(**A**) In-line transmission spectra for pure spinel samples prepared by laser direct deposition (with sample thickness of 1.9 mm) compared with sintered counterparts with similar composition (*n* = 1.5) [13]; (**B**) Infrared in-line transmittance for pure spinel samples prepared by laser direct deposition and sintering [55]; (**C**) The effect of SiO_2_ percentages on the total transmittance of the SiO_2_ doped spinel samples fabricated by laser direct deposition.

The measured in-line transmission spectra for pure spinel samples (0 wt.% silica) are summarized in Figure 6A near visible range and Figure 6B near infrared range. The measured transmission values were relatively lower than those of sintered counterparts [13,55] due to a relatively higher porosity as measured in 3D printed pure spinel samples in Section 3.2.3. In the meantime, the in-line transmission was found to severely deteriorate due to dramatically increased light scattering for all silica doped spinel samples. Thus, the total transmittance was also measured to study the effect of silica dopant percentages on doped spinel samples as summarized in Figure 6C. As expected, the highest total transmission value was measured at 82% for the pure spinel samples (0 wt.% silica). The addition of 0.5 wt.% silica dopants lowered the obtained total transmission to 52%. Further increasing silica dopant percentages dramatically reduced the total transmission down below 24%. The decreased transmission values with the addition of silica dopants are believed to be related to the increased porosity in Section 3.2.3 and the formation of secondary phase characterized in Section 3.2.2. It should also be noted that the optical transmission needs to be improved for applications of the printed spinel ceramics. A short duration in a hot isostatic press was found to be beneficial in removing residual defects in laser deposited nickel-based super alloys [56], thus worth further detailed investigation of similar post-processing techniques on the printed spinel samples to improve obtained optical transparency.

### 3.2. Microstructural and Compositional Characterization

**Figure 7:**
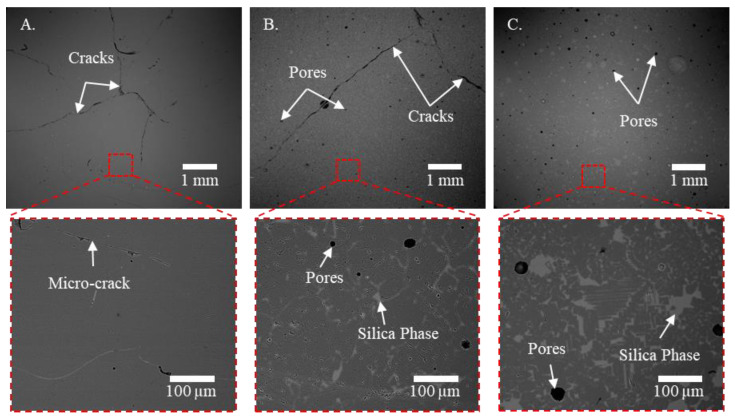
Backscattered electron SEM images of typical polished spinel samples printed at a laser power of 580 W with silica doping percentages of (**A**) 0 wt.%, (**B**) 5 wt.%, and (**C**) 10 wt.%. The inset images show close-up views of the obtained microstructure with presence of micro-cracks for pure spinel samples and silica phases after the addition of silica dopants.

The typical microstructural characteristics of the printed spinel samples are shown in Figure 7. Compared with the silica doped samples, more cracks were found in the pure spinel samples. A close-up view showed the presence of micro-cracks along grain boundaries, which were not visible at the macro-scale but would increase light scattering [57]. Meanwhile, more pores were found after increasing silica doping percentage. A silica phase was also observed within all silica doped spine samples. Thus, XRD and EDS analyses were further performed to examine the composition of these phases and correlate with doped silica. It is worth noting that a seemingly refined microstructure was found in doped samples, as further confirmed by measured grain size below.

#### 3.2.1. X-ray Diffraction Characterization

**Figure 8:**
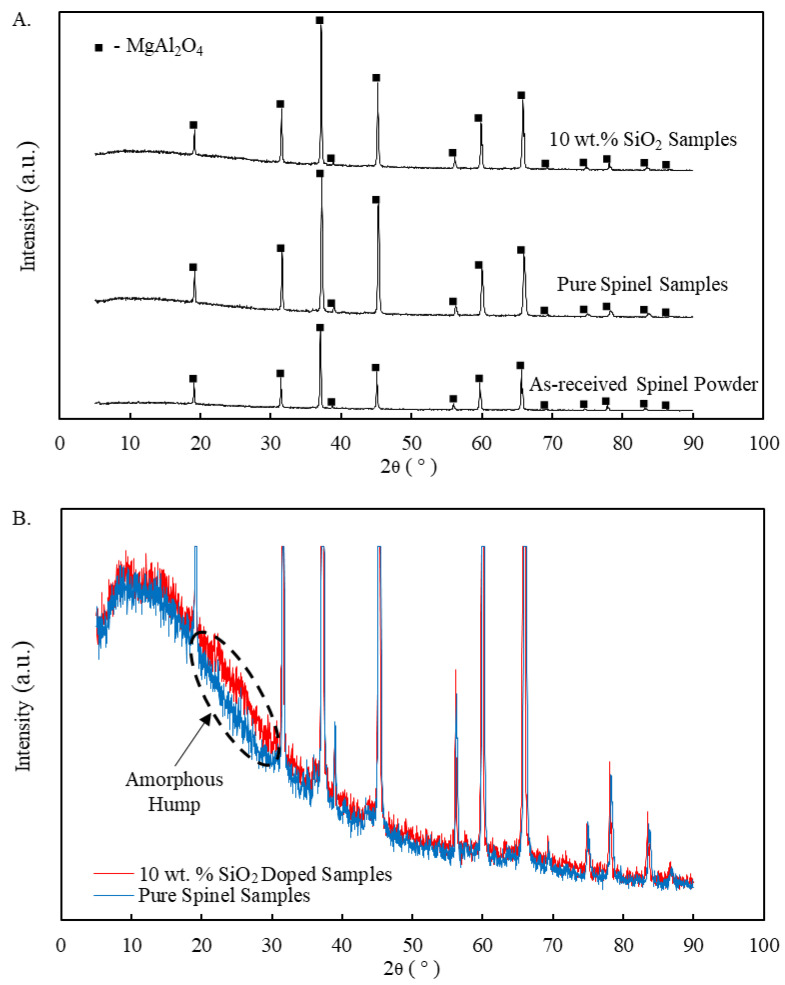
XRD peak patterns for printed pure and 10 wt.% SiO_2_ doped spinel samples with (**A**) in comparison with the as-received spinel powders, and (**B**) highlighting the detected silica amorphous hump.

XRD patterns were measured for the printed spinel samples the as-received spinel powders. A comparative peak analysis for pure and 10 wt.% silica doped spinel samples is shown in Figure 8A. The only detected phase for all printed samples (with or without silica dopant) was crystalline spinel. It is worth noting that since the alumina-rich spinel (MgO·1.4Al_2_O_3_) powders were used in this study, a slight peak shift was observed for all samples compared to the standard for spinel (MgAl_2_O_4_) (PDF # 01-073-1959). A shift to larger 2θ angles for MgAl_2_O_4_ solid solution corresponds to higher amounts of alumina (indicating larger *n* values in the chemical formula MgO·nAl_2_O_3_) [58]. Interestingly, all printed spinel samples showed even larger 2θ angles compared to those of as-received spinel powders, indicating an alumina-richer spinel phase produced within the printed samples. It could be attributed to the composition fluctuation due to different vapor pressures of MgO and Al_2_O_3_ [59] during melting process for the proposed laser direct deposition method.

XRD results in Figure 8 indicated the presence of single spinel phase even for the silica doped samples. The added silica is expected to mainly form an amorphous phase as no additional peaks were detected but only with the presence of an amorphous hump at 2θ angles from approximately 20 to 30 degrees (highlighted in Figure 8B). It should be noted that the intensity of the highest peaks in Figure 8B were cut to more clearly illustrate the amorphous hump observed for silica doped samples. Amorphous silica is typically identified as a broad hump (increase) in detected XRD intensity for 2θ angles from around 15 to 30 degrees [60]. Similar trends in both 2θ shift and amorphous hump were also observed for all other samples doped with different percentages of silica in this study.

#### 3.2.2. EDS Characterization

**Figure 9:**
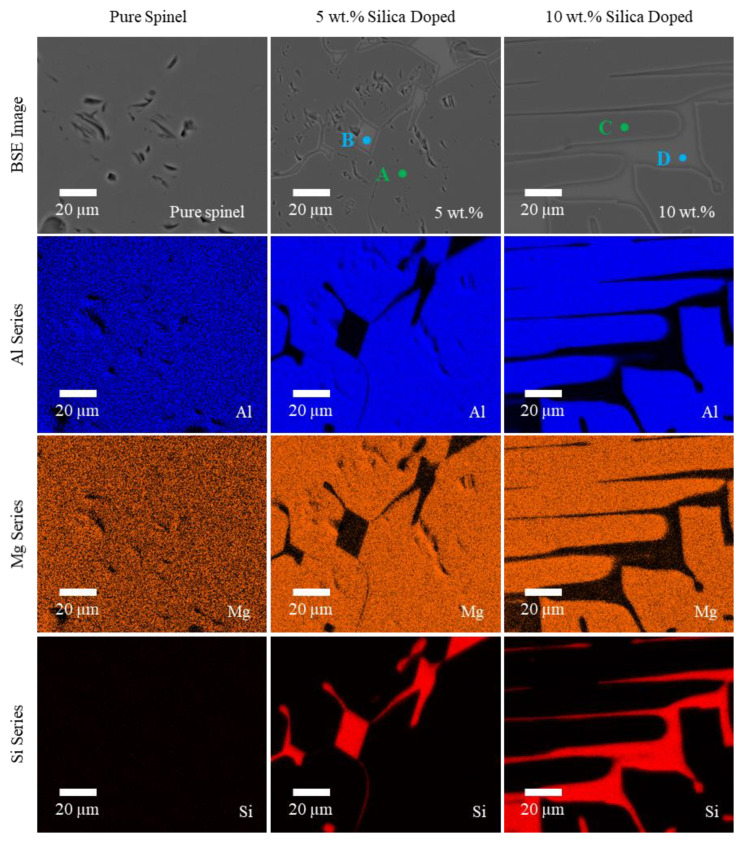
Backscattered electron SEM images and EDS elemental maps showing the primary composition of pure, 5 wt.% and 10 wt.% silica doped spinel samples printed at 580 W. The highlighted A–D indicate the locations for further elemental analysis of different phases.

Elemental maps for primary composition of pure, 5 wt.% and 10 wt.% doped spinel samples are shown in Figure 9. As expected, primary elements in all samples were aluminum, magnesium, and oxygen, while silica doped samples included additional silicon. The pure spinel samples showed a nearly homogeneous mixture of primary elements across the whole area. On the other hand, two distinct phases, i.e., silica phases dispersed within spinel phases, were observed on the silica doped samples.

The elements of both phases were further analyzed by P/B-ZAF, an EDS analysis technique [61] to obtain absolute concentration values. The specific locations of analysis have been highlighted for the doped spinel samples in Figure 9. A and C both showed the spinel phases while B and D showed the silica phases in 5 wt.% and 10 wt.% doped samples, respectively. It should be noted that as only a single spinel phase existed for the pure spinel samples, the whole area was used for detection and analysis. From the results summarized in Table 1, the compositional distribution of the spinel phases were largely consistent for the detected primary elements (for pure samples as well as A and C in the doped samples), corresponding to approximately *n* = 1.6 by average for MgO·nAl_2_O_3_. It was larger than *n* = 1.4 from the as-received spinel powders and explained the XRD peak shift observed in Figure 8 from stoichiometric spinel as well as the as-received spinel powders. No residual silicon was observed in the spinel phases. Meanwhile, the spinel phases exhibited a slight oxygen deficiency (about 8.3 mol%). This is attributed to oxygen vacancy formation during laser melting, which is believed to result in the yellowish color seen in the printed spinel samples in Figure 4. Similar results were also observed in additively manufactured yttria stabilized zirconia (YSZ) ceramics [62]. It is expected that oxygen content may be restored through a post annealing process.

As shown in Table 1, similar compositions were found in the silica phases (B and D) for silica doped samples. It is worth noting that the slight amount of calcium observed came from the impurities of as-received silica powders. Despite the presence of aluminum and magnesium, the silica phases were expected to be amorphous as detected above from the XRD results. The formed secondary phases may exhibit a refractive index different than that of spinel, thus increasing light scattering and lowering optical transmission. Meanwhile, due to the addition of softer silica with lower CTE, the softer secondary phases dispersed within the spinel phases may lower thermal stress and reduce crack formation as further investigated below.

#### 3.2.3. Porosity Characterization

**Figure 10:**
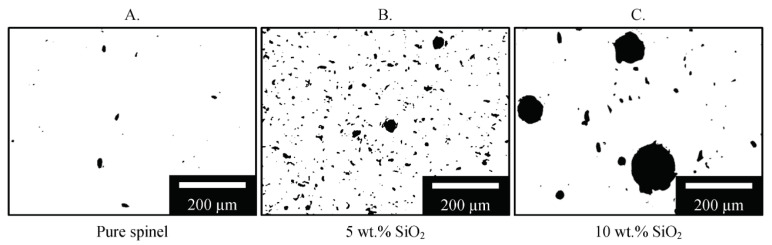
Processed optical micrographs for the porosity analysis of spinel samples printed at a laser power of 580 W with (**A**) pure spinel, (**B**) 5 wt.% SiO_2_, and (**C**)10 wt.% SiO_2_.

Achieving near full densification is one of the key requirements in fabrication of highly transparent spinel ceramics [63,64], which is typically related to residual porosity within fabricated samples. The effect of silica dopants on porosity was thus characterized. The processed images in Figure 10 were obtained by binarizing/thresholding of optical micrographs to only show pores to facilitate porosity measurement.

**Figure 11:**
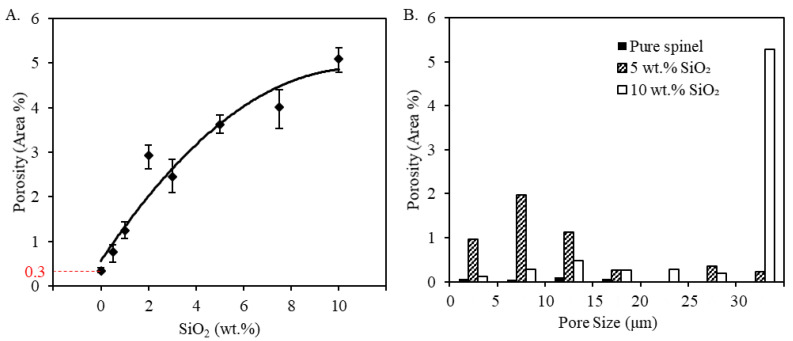
Porosity analysis of spinel samples with silica dopants printed at a laser power of 580 W: (**A**) shows overall porosity fraction with respect to doping percentages, and (**B**) shows typical pore size distribution obtained at different doping compositions.

As shown in Figure 11A, a minimum porosity of 0.3% was measured in the printed pure spinel samples (0 wt.%), yielding highly transparent samples in Figure 4. On the other hand, the addition of silica increased both the number and size of pores within the printed samples as shown in Figure 10, resulting in higher porosity measured in Figure 11A. Porosity increased from 0.3% to 5.1% after silica doping contents increased from 0 wt.% and 10 wt.%, which is expected to be a major contributing factor in the greatly reduced optical transmission observed in Figure 5 due to drastically increased light scattering. A similar trend was also recently found during laser direct deposition of nontransparent alumina ceramics [33], where the measured relative density reduced due to increased porosity after the addition of TiO_2_ dopants.

The increased porosity is attributed to a vapor pressure difference between spinel and silica when heated to or above the melting temperature [59], which resulted in a higher degree of gas porosity inclusion. The melting point of silica (1710 °C) [65] is much lower than that of spinel (2135 °C) [17], and the boiling point of silica (2230 °C) [66] only about 100 °C higher than the boiling point of spinel. Due to the relatively small difference between the melting point of silica and the boiling point of spinel, the high laser irradiance necessary to melt spinel and remove porosity was sufficient to vaporize silica and introduce new gas bubbles in the melt. If solidification occurs before removal of these newly created gas bubbles, there will be an increase in residual porosity. Moreover, addition of silica is expected to modify the viscosity of the melt [66], potentially increasing residual porosity and altering the pore size distribution shown in Figure 11B. The pore size distribution is presented in a weighted histogram where the area of each pore was first measured, and then converted into circular diameter equivalents [52], and normalized by the analyzed cross-sectional area. In other words, each bar represents the pore area fraction for each size range. A near uniform pore size distribution was observed for pure spinel samples. The addition of 5 wt.% silica mainly increased the percentage of small pores with an equivalent diameter less than 20 µm. On the other hand, at 10 wt.% silica there was a significant shift to larger pore sizes greater than 30 µm, while simultaneously decreasing the number of smaller pores. This is believed to be mainly attributed to the combination of small pores into large pores, as seen in Figure 10C.

#### 3.2.4. Grain Size Characterization

**Figure 12:**
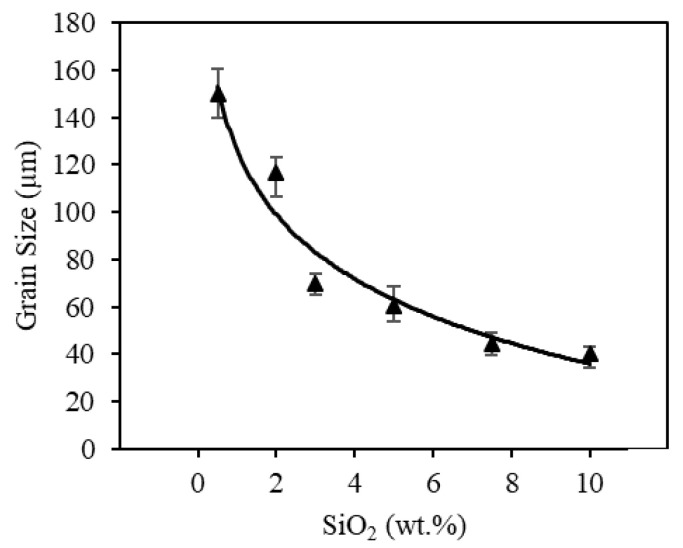
Effect of silica doping on grain size of the printed spinel samples.

**Figure 13:**
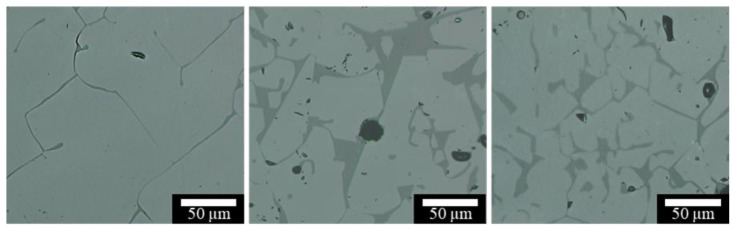
Typical optical micrographs of spinel samples with (**A**) 0.5 wt.% SiO_2_, (**B**) 5 wt.% SiO_2_, and (**C**) 10 wt.% SiO_2_. Obvious grain refinement was observed after increasing SiO_2_ dopant percentages.

Previous studies [33,37] suggested that dopants possibly refine the obtained microstructure and thus strengthen the printed nontransparent alumina ceramic samples. Thus, the average grain size was also measured to study the effect of silica dopants on the printed spinel samples. As summarized in Figure 12 and demonstrated in Figure 13, after increasing the silica percentage, the grain size continuously decreased, with a minimum average grain size of 40 µm measured for 10 wt.% silica doped samples.

Reduction in grain size with an increase in silica can be attributed to two primary factors: increased nucleation via constitutional supercooling and reduced grain boundary mobility. Constitutional supercooling occurs during solidification when a mixture contains a component that is insoluble below the solidus temperature. In this case, during solidification and grain growth, the solute is rejected to the liquid at the solid/liquid interface resulting in a concentration of the solute, which leads to a reduction in the liquidus temperature for the element that partitions to the liquid [67]. This reduction in liquidus temperature results in a liquidus temperature gradient near the solid liquid interface. The actual temperature of the liquid just ahead of the developing grain can dip below the liquidus temperature, creating a condition conducive of homogeneous nucleation. When constitutional supercooling causes sufficient undercooling required for nucleation of potent particles, equiaxed grains will nucleate [68]. Since constitutional supercooling is dependent on the amount of solute present, more homogeneous nucleation of equiaxed grains may occur at higher silica percentages, partially explaining the large reduction in grain size observed here. On the other hand, impurity segregation to the liquid near the solid/liquid interface also affects grain boundary mobility in spinel ceramics [69]. The solute segregation hinders grain boundary mobility with increased resistance [70], thus yielding lower grain growth rate and smaller grains.

#### 3.2.5. Crack Characterization

**Figure 14:**
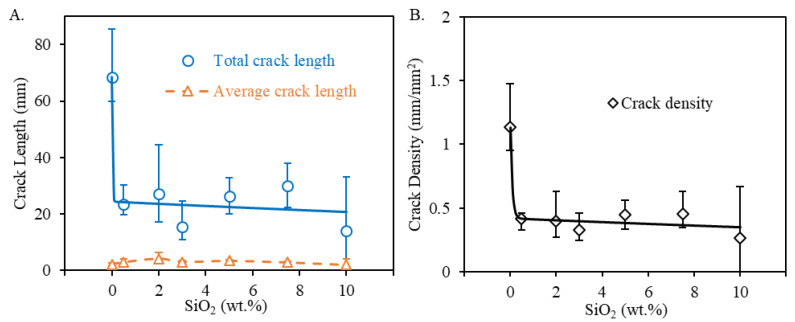
Results for processing induced cracking: (**A**) shows total crack length and average crack length, and (**B**) shows crack density measured for spinel samples printed at a laser power of 580 W with varying silica doping percentages.

As seen in Figure 4 and Figure 5, processing-induced cracking was a major limiting factor in the printed transparent spinel ceramics while doping apparently showed promising results in crack reduction. Thus, it is necessary to characterize crack formation in terms of silica dopants used here. Total crack length, average crack length, and crack density were measured for each doping composition shown in Figure 14. Four different printed samples were measured for each composition.

In accordance with the observations in Figure 5, silica doping reduced crack formation, particularly the total crack length in Figure 14A and the crack density in Figure 14B, showing its feasibility in crack control for laser direct deposited spinel ceramics. The addition of merely 0.5 wt.% silica contents drastically decreased both total crack length and crack density. Increased silica dopants reduced average total crack length by up to 79% and average crack density by up to 71%. However, further increasing silica dopants in general only exhibited a moderate reduction trend in cracking. It is also worth noting that a similar trend in crack reduction was also observed when using TiO_2_ as dopants during laser direct deposition of nontransparent alumina ceramics [30]. The consistent findings suggested that a low doping level may be sufficient to achieve an efficient crack reduction control. This is particularly necessary in consideration of the deteriorating effect of high doping percentages observed in Figure 5 on optical transmission. It will thus be necessary to study doping mechanisms in crack control so that a minimal level of dopants can be used in order to efficiently minimize crack formation within the printed transparent samples. It is worth noting that the variation in the reduction of total crack length and crack density in Figure 14 could be attributed to the doping variation due to silica vaporization during deposition as well as increased porosity as shown above.

Interestingly, no obvious reduction in the average crack length was observed after the addition of silica dopants. The measured average crack length within the pure spinel samples was nearly comparable to those of the doped spinel samples. The obvious reduction in total crack length and crack density seen above was thus mainly attributed to the reduction in the number of cracks. It showed that silica doping was only able to inhibit crack initiation but not crack propagation.

During laser direct deposition process, high cooling rate (up to 10^6^ K/s [66]) during cooling and solidification process after deposition results in high thermal gradient and stress that may lead to thermal shock. The maximum temperature that a material can withstand without initiation of cracks is defined as the thermal shock parameter (R) [71,72]

(1) $$R=\frac{\sigma \left(1-\upsilon \right)}{E\alpha },$$

where E and υ denote Young’s modulus and Poisson’s ratio, respectively. α represents CTE, and σ shows the strength of the material. The elastic moduli of silica and spinel are 74 GPa [73] and 295 GPa [18], respectively. The CTEs are 0.6 × 10^−6^/K and 9.0 × 10^−6^/K for silica [73] and spinel [18], respectively. The silica doped secondary phase found above is expected to have lower CTE and modulus compared to the spinel phase, thus reducing the overall CTE and modulus of the doped spinel samples in Equation (1). Meanwhile, the spinel strength typically increases [18] with a decreasing grain size as observed above after doping. Thus, the addition of silica is predicted to increase the thermal shock parameter in Equation (1) and improve the resistance to crack initiation.

On the other hand, further increasing silica dopants may keep lowering the effective CTE and modulus of the doped samples. However, the effective strength of the doped spinel will be gradually weakened by higher percentages of weaker silica [73], lowering doping benefits in further crack reduction as observed in Figure 14. Meanwhile, as shown in Table 1, similar compositions were found in the secondary phases for the silica doped spinel samples. Once cracks are nucleated, the similar mechanical strength of these secondary phases, regardless of silica doping percentages, is believed to yield no obvious effects on inhibiting crack propagation.

### 3.3. Mechanical Characterization

#### 3.3.1. Microhardness

**Figure 15:**
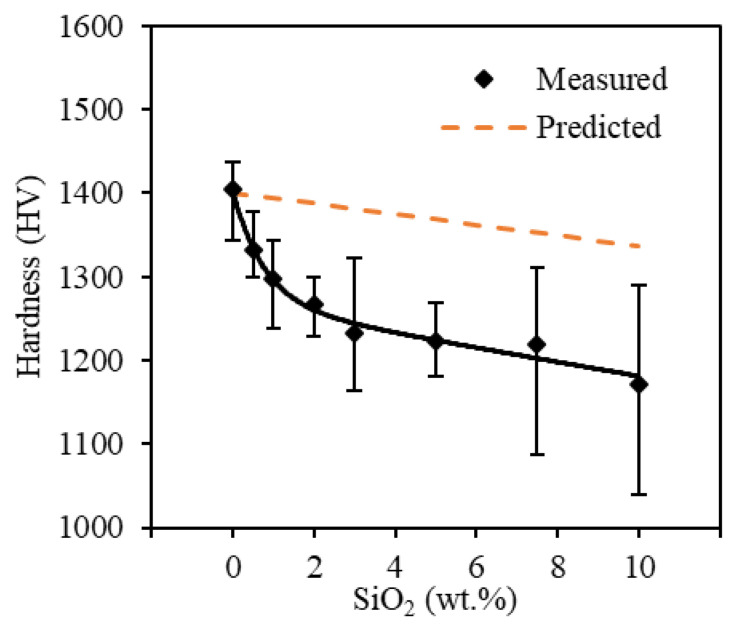
Microhardness of printed spinel samples with added silica ranging from 0 wt.% to 10 wt.% in comparison with predicted values by a volumetric rule of mixtures.

The measured microhardness for the printed spinel samples is summarized in Figure 15. The obtained hardness value for the printed pure spinel was around 1400 HV, nearly comparable to that of sintered counterparts [15]. Meanwhile, the microhardness of doped samples steadily decreased with an increase in silica contents from 0 to 10 wt.%. Young’s modulus (E) can be approximated using E≅20·H [33]. The lower hardness (H) after doping corresponded to a reduction in the effective modulus (E) in Equation (1), which helped increase the resistance of the doped spinel samples to crack initiation as discussed above.

As the doped silica formed secondary phases dispersed within spinel phases shown in Figure 7, the hardness of the doped spinel samples (H) can be predicted based a volumetric rule of mixtures [74] as

(2) $$H=f_{spinel}H_{spinel}+f_{SiO_{2}}H_{SiO_{2}},$$

where $f_{spinel}$ and $f_{SiO_{2}}$ are the volume fractions of spinel and silica, respectively. $H_{spinel}$ and $H_{SiO_{2}}$ are the theoretical hardness values of spinel and silica, respectively. The properties of silica were used here to model the properties of the secondary phases. Pure spinel ceramics have microhardness ranging between 1300–1400 HV, depending on post-processing and grain sizes of the produced spinel ceramics [15]. In comparison, the hardness of silica is much lower at approximately 650 HV [75]. As the percentage of softer silica dopants increased, the predicted hardness of doped spinel ceramics decreased in Figure 15. However, the measured hardness values became increasingly lower than those predicted by the rule of mixtures at higher doping percentages. The discrepancies can be attributed to the fact that the predicted values were calculated based on the silica hardness values. However, as discussed above, the doped silica instead formed a newer amorphous secondary phase, the hardness of which may be different than that of silica. Meanwhile, the increased porosity at higher doping percentages found above would also reduce the overall hardness of the doped spinel ceramics, thus yielding a lower hardness value than the prediction results.

#### 3.3.2. Fracture Toughness

**Figure 16:**
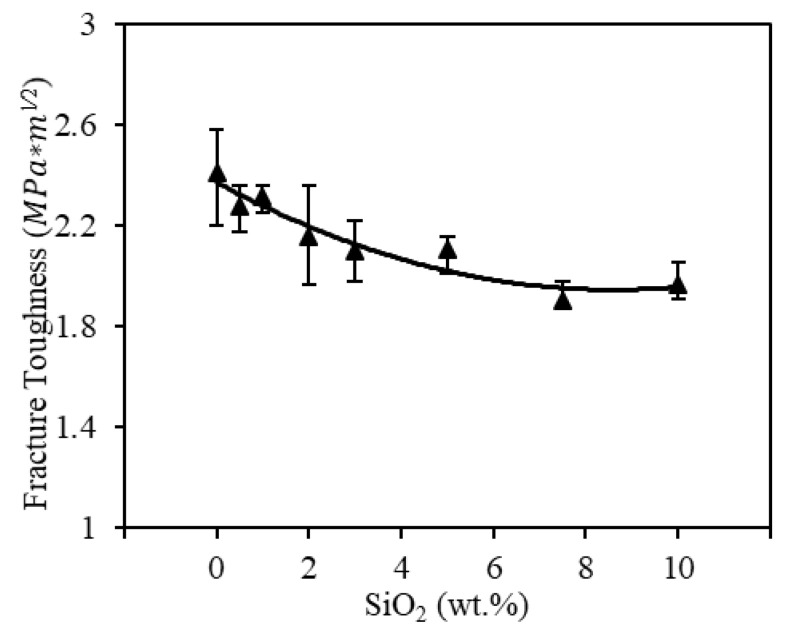
Fracture toughness results for the printed spinel samples with doped silica ranging from 0 wt.% to 10 wt.%.

**Figure 17:**
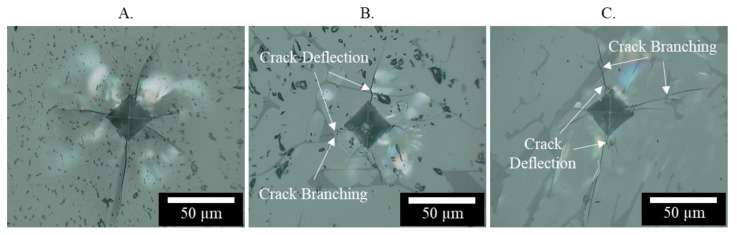
Typical indentation crack patterns for (**A**) pure, (**B**) 5 wt.%, and (**C**) 10 wt.% silica doped spinel samples.

While the thermal shock parameter (R) affects crack initiation, fracture toughness will affect crack propagation within the printed spinel samples. Thus, the fracture toughness for both pure spinel and silica doped samples was measured. The length (l) of Palmqvist cracks induced by Vickers indentation on the polished samples was first measured. The fracture toughness ($K_{IC}$) was then calculated as [76]

(3) $$\left(\frac{K_{IC}\cdot \emptyset }{Ha^{1/2}}\right)\cdot {\left(\frac{H}{E\cdot \emptyset }\right)}^{\frac{2}{5}}=0.035\cdot {\left(\frac{l}{a}\right)}^{-3/2},$$

where ∅≅3 is a constant. a denotes the half diagonal of the indentation. The hardness (H) data measured above was used. The Young’s modulus (E) was approximated as E≅20·H [33]. As shown in Figure 16, highest average fracture toughness were obtained from the printed pure spinel samples (0 wt.%), at an average of 2.4 ${\mathrm{MPa}*m}^{1/2}$ and similar to those reported for conventionally fabricated spinel ceramics (typically ranging from 1.4 to 2.2 ${\mathrm{MPa}*m}^{1/2}$ [14,42]). The indent morphology in Figure 17A was also found to be similar to that of conventionally prepared (hot pressed) spinel ceramics [77].

Further increasing silica doping percentages instead lowered the fracture toughness of the printed spine samples to an average of 1.9 *MPa∗m*^1∕2^ with the addition of 10 wt.% silica. As observed in Figure 17, the silica dopants indeed led to crack branching and crack deflection within the doped spinel samples under indentation, both of which are beneficial to increase the fracture surface energy ($\gamma^{*}$) in Equation (4). However, such benefits on crack propagation are outweighed by the reduction of the modulus (E) in Equation (4) due to the formation of silica doped secondary phase with lower modulus, thus leading to a decrease in actual fracture toughness. The decreasing fracture toughness, related to crack propagation, also helped explain why no obvious change in the average crack length was observed at a higher doping percentage. This further showed the potentials of a low doping level in more efficiently controlling crack formation. It should be noted that the nearly circular dark phase in Figure 14 corresponded to observed pores.

(4) $$K_{IC}=\sqrt{2E\gamma^{*}}.$$

## 4. Conclusions

This study demonstrated the feasibility of 3D printing transparent spinel ceramics via a laser direct deposition process. Highly transparent spinel ceramics with the highest total optical transmittance of 82% at a wavelength of 632.8 nm, and with a minimum porosity of 0.3% were obtained by the proposed 3D printing method. The primary issue for the fabricated transparent spinel ceramics was prevalent cracking. It was shown that with silica doping, the average total crack length was reduced by up to 79% and the average crack density was reduced by up to 71% due to refined microstructure and the formation of softer secondary phase with lower CTE. However, higher doping percentage showed no additional significant benefits in crack reduction but severely lowered the obtained optical transmission due to an increased porosity and the formed secondary amorphous phase. It showed the necessity of using a minimal amount of doping to both efficiently control crack formation and obtain high transparency. The average microhardness decreased from 1400 HV to 1170 HV and average fracture toughness decreased from 2.4 *MPa·m*^1∕2^ to 1.9 *MPa·m*^1∕2^ after increasing silica contents from 0 wt.% to 10 wt.%. These well demonstrated the potentials of the proposed AM method in fabrication of transparent ceramic components with dopants and are thus worth future investigations.

## Figures and Tables

**Table 1:** Comparison of elemental compositions for the pure and silica doped spinel samples.

	Locations	Al (wt.%)	O (wt.%)	Mg (wt.%)	Si (wt.%)	Ca (wt.%)
Pure spinel	-	45.4	43.7	10.9	0.0	0.0
5 wt.% doped	A	43.6	43.2	13.2	0.0	0.0
	B	20.5	44.5	8.5	24.5	2.0
10 wt.% doped	C	44.2	43.8	12.0	0.0	0.0
	D	16.1	46.1	7.0	29.2	1.6
